# Removal of intraconal bullet through endoscopic transnasal surgery with image-guided navigation system 8 months after injury: a case report

**DOI:** 10.1186/s13256-019-2007-x

**Published:** 2019-03-17

**Authors:** Chakapan Promsopa, Usaporn Prapaisit

**Affiliations:** Division of Allergy and Rhinology, Department of Otolaryngology Head and Neck Surgery, Faculty of Medicine, Prince of Songkla University, Hat Yai, Songkla 90110 Thailand

**Keywords:** Bullet, Transnasal endoscopic approach, Navigator-assistance, Orbit, Case report

## Abstract

**Background:**

Lodgment of a bullet within the orbit is uncommon. The decision to remove these objects poses a challenge to surgeons due to a high risk of complications. Currently, endoscopic transnasal surgery with navigator assistance facilitates the localization of foreign bodies allowing their safe removal with minimal surrounding tissue damage or optic nerve injury.

**Case presentation:**

We describe a case of a 26-year-old Thai woman with a chronic intraorbital foreign body located within her medial intraconal space. The chronic intraorbital foreign body was successfully removed by endoscopic transnasal surgery, combined with assistance from a navigation system, 8 months after injury without any damage to her eye or disturbance in vision.

**Conclusion:**

Intraconal foreign bodies, such as bullets, are a chronic problem and should be observed in the long term; prompt surgical removal should be performed if indicated.

## Introduction

Bullet injuries to the face are uncommon. They are dangerous due to the complexity of craniofacial anatomy and the presence of vital structures. The retrieval of intraorbital foreign bodies is technically difficult and challenging. Classically, external approaches have been the most widely used; however, these are invasive and associated with several major disadvantages, such as postsurgical scarring and considerable morbidity [1]. Recently, improvement in technology and our understanding of anatomy have gradually progressed to enable minimally invasive procedures, such as endoscopic surgery. Endoscopic surgery has the advantage of gaining access, transnasally, into the medial intraconal space with minimal surrounding tissue damage and with no unsightly external scars [2]. This case report presents a case of a 26-year-old woman who had an accidental gunshot injury in which the bullet was retained in her left eye; the gunshot injury developed into eye pain 8 months after the injury. Fortunately, the bullet was successfully removed via a transnasal endoscopic approach with the aid of an image-guided navigation system, without any morbidity.

## Case presentation

**Fig. 1:**
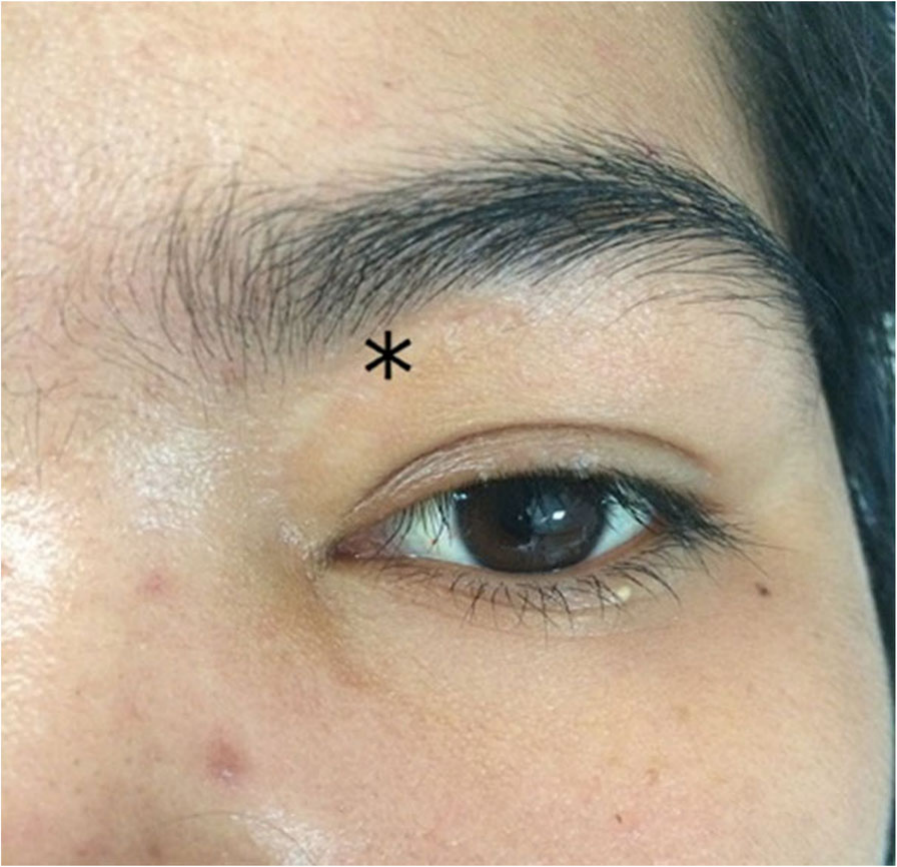
*Asterisk* shows minimal scar wound, at the point of foreign body entry, located superomedially of left upper eyelid

**Fig. 2:**
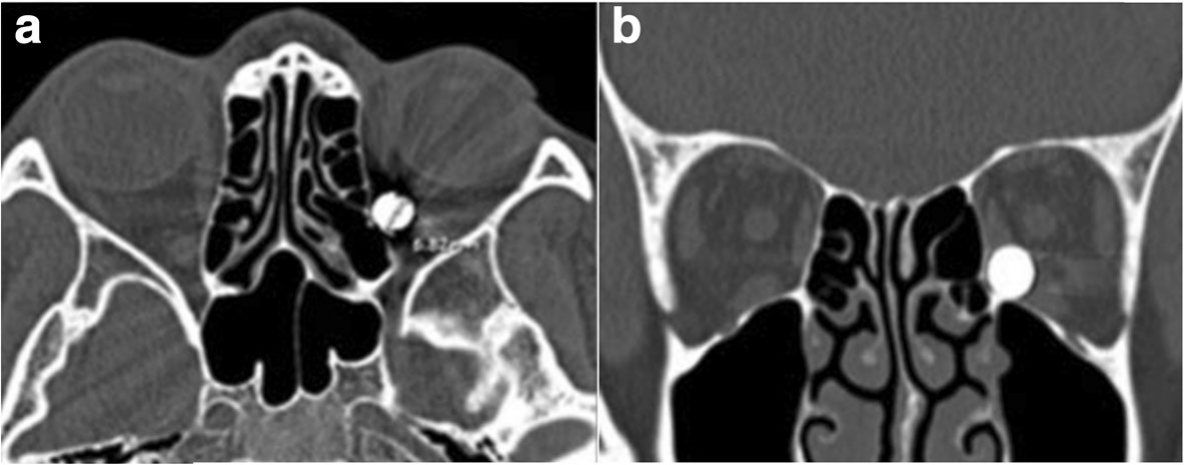
An axial (**a**) and coronal (**b**) cut section of a computed tomography scan image of the left orbit shows a round-shaped metallic foreign body embedded in the medial intraconal space lying lateral to posterior ethmoid sinus

**Fig. 3:**
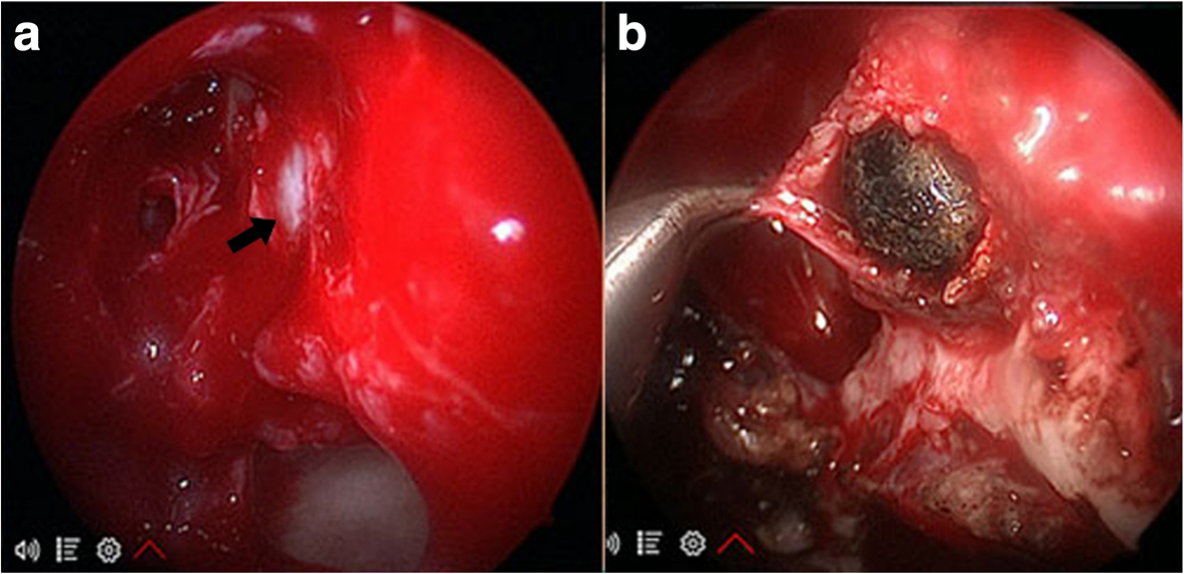
**a** Left transnasal endoscopic view of completed middle meatal antrostomy with anterior-posterior ethmoidectomy and the bullet located in the lamina papyracea (*black arrow*). **b** The fibrotically encapsulated bullet was found lying in the orbital fat after part of the lamina papyracea was removed and the periorbita incision was done

**Fig. 4:**
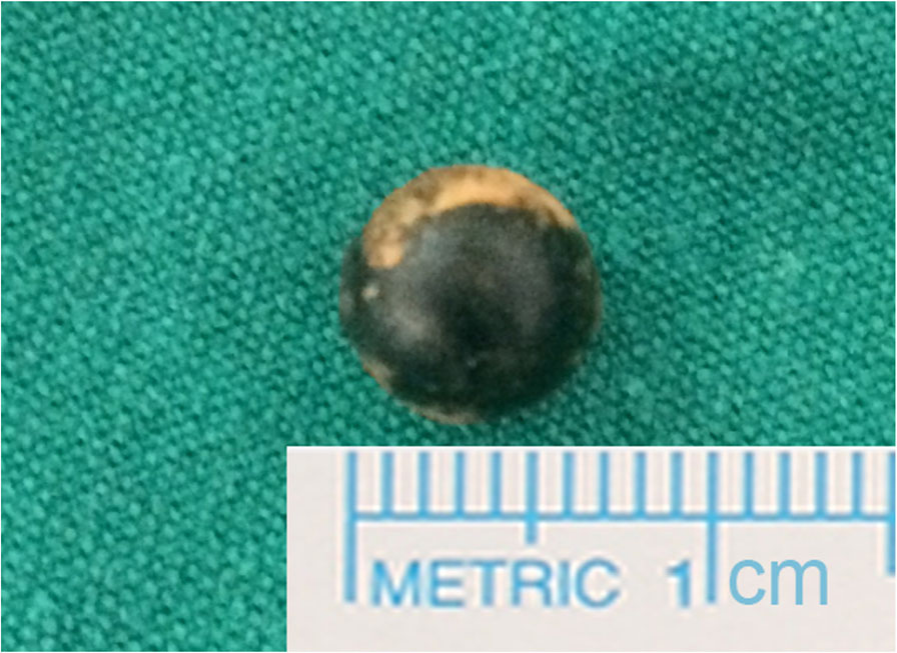
The retrieved metallic foreign body measured 6 mm

A 26-year-old Thai woman presented with a foreign body in her left orbit that had been retained for 8 months. Eight months previously, she had sustained a gunshot injury to her left eye. There was only a small wound on her left eyelid (Fig. 1); she had normal eye movement, a normal visual field, and no pain. A plain radiograph was performed at that time; it detected a round-shaped metallic foreign body located medially in her left eye globe. She was then diagnosed as having a retained foreign body in her left orbit and she was advised to have clinical observation. However, 8 months later, she developed pain in her left eye without any sinonasal symptoms. After discussion about the risk of surgery and retention of an orbital foreign body, an ophthalmologist referred her to our department for the minimally invasive procedure option of removal of the foreign body using an endoscopic transnasal approach. On examination, the movements of her left orbit were not restricted and there was normal visual acuity (20/20) with no proptosis or chemosis. A computed tomography (CT) scan of her left orbit revealed a round-shaped metallic foreign body in the medial intraconal space, and lateral attachment of posterior ethmoid sinus, measuring 6 mm (Fig. 2). A transnasal endoscopic approach, with navigator assistance (Brainlab), was used to remove the bullet. Uncinectomy and anterior-posterior ethmoidectomy were performed. The location of the intraconal metallic foreign body was confirmed with a navigator system, then part of the lamina papyracea was removed and the periorbita incision was done. The defect was enlarged and the fibrotically encapsulated bullet was found lying in the orbital fat (Fig. 3). The fibrotic capsule was dissected, and the bullet was delivered through her left nostril with probes and curetted (Fig. 4). No intraorbital bleeding or damage to any soft ocular structures was noted. A relative afferent pupillary defect was found in her left eye during removal of the foreign body, but it resolved and no postoperative complications were observed.

## Discussion

Gunshot injuries to the craniofacial region are uncommon but can cause loss of life or irreversible damage to vital organs. The bullet may traverse in any direction and/or lodge in any site of the craniofacial region; the commonest site of lodgment of facial foreign bodies is the paranasal sinuses [3–6]. It is rare for a bullet to be lodged in the orbital cavity without causing much damage to the orbital structure, as seen in the present case. From an anatomic point of view, the orbit is a highly complex area which is divided into two compartments by the extraocular muscles: intraconal and extraconal [7]. Traumatic intraocular foreign body injury can be associated with partial or complete loss of visual function. A tiny foreign body retained within the orbit can cause either immediate or delayed complications, including chronic orbital inflammation, osteomyelitis, thrombotic vasculitis, or diffuse infections from septicopyemia. Although these injuries often lead to serious consequences, in some cases they may have a good long-term prognosis. A chart review study of 43 patients with metallic orbital foreign bodies that were retained from 6 months to 63 years (median, 2 years) found that they were generally well tolerated [8], as with our patient who did not have any symptom until 8 months later.

Optic nerve injury results in both mechanical and ischemic damage. Walsh divided this damage into primary or secondary mechanisms [9]. Primary mechanisms result in permanent injury to the optic nerve axons at the moment of impact. Secondary mechanisms include vasospasm and swelling of the optic nerve, within the boundaries of the optic canal, leading to the worsening of ischemia and further loss of axons in the period following the trauma [9].

Radiology studies are important initial tests; plain radiology is helpful to confirm diagnosis and localize the foreign body. However, a CT scan helps pinpoint the exact location of the bullet; hence, providing a roadmap for safe and precise removal. Although magnetic resonance imaging (MRI) provides very detailed soft tissue architecture, it is contraindicated in cases of metallic foreign bodies because of the potential risk for migration and further injury.

Indication for the removal of an orbital foreign body is always to be decided upon individually, the physician must weigh the risk of surgery against the risks of retention, including fistula formation or infection [10]. Surgery is particularly indicated if there are acute or chronic functional restrictions, or inflammatory reactions which are summarized in Table 1 [11].

**Table 1:** Indications for surgical removal in patients with intraorbital foreign bodies

Indications	
Palpable orbital mass	
Signs of orbital infection or inflammation	
Orbital symptoms: pain, proptosis, decreased visual acuity, and restricted mobility	
Optic nerve compression	
Large or sharp-edged foreign body	
Suspicion of inorganic foreign bodies or copper materials	

The surgical approach, for removal, depends on the nature of the body, its location (anterior or posterior orbit), and associated complications (infections, optic nerve lesions or compression, and lesions to the extraocular nerve or intraorbital blood vessels) [12]. Conventional open methods of removal increase morbidity, scarring, disfigurement, and other complications. Transnasal endoscopic removal is safe, less damaging, and easy because it gives you direct visualization. Endoscopic removal of a bullet from the orbit has been reported in the literature [13–15]. Furthermore, the navigation system has been shown to be an essential element, working in combination with endoscopic intervention, for precise location of the target, thereby enabling surgeons to make the smallest possible opening in the bone and periorbita [16, 17]; so, transnasal endoscopic surgery is becoming increasingly popular as a safe surgical technique to access the medial intraconal space.

In the present case, the bullet was removed via a transnasal endoscopic approach through anterior and posterior ethmoid sinus from the intraconal area. This approach is minimally invasive and recommended in cases of foreign bodies, especially in the midline craniofacial region, in combination with an image-guided navigator system, which greatly enhances the chances of surgical success with minimal ocular complications.

## Conclusion

Intraconal foreign bodies, such as a bullet, are a chronic problem and should be observed in the long term, so as to perform prompt surgical removal if indicated. Endoscopic transnasal surgery, with navigation system assistance, is a safer and less invasive approach than classic surgical techniques. Currently, a navigation-assisted surgery system has been shown to be an essential element in endoscopic intervention, which facilitates a trend in endoscopic intervention becoming more available.
